# Supplementary material for: ATG101 Degradation by HUWE1-Mediated Ubiquitination Impairs Autophagy and Reduces Survival in Cancer Cells
Source: Int J Mol Sci. 2021 Aug 25;22(17):9182. doi: 10.3390/ijms22179182 (PMC8430637; doi:10.3390/ijms22179182)
Supplement: Supplementary file 1 [file ijms-22-09182-s001.zip › IJMS1299763_Supplementary materials_legend.pdf]

Supplementary materials legends

Table S1. E3 ligase candidates among ATG101-interacting proteins. E3 ligases potentially ubiquitinating ATG101 in MIA PaCa-2 cells were identified by LC/MS after immunoprecipitation of ATG101 FL or ΔC ATG101.

Figure S1. Reduction of mitophagy by ATG101 knockdown is assessed by using mt-Keima. HeLa cells stably expressing mt-Keima and Parkin were transfected with ATG101 siRNA (siATG101) or scramble siRNA (siCTL) for 24–48 h. Cells were treated with CCCP (10 μM) for 4 h prior to image acquisition by confocal microscopy. At least five distinct regions were imaged per condition. Scale bar: 20 μm.

Figure S2. Ubiquitination levels of ATG101 in starvation condition were comparable to the those in nutrient basal condition. (a) HEK293T cells were transfected with HA-Ubiquitin (Ub) and/or FLAG-ATG101, and HA-Ub for 24–48 h, then incubated in nutrient complete medium or amino acid deprivation medium (−AA) for 4 h prior to immunoprecipitation analysis. Then these cell lysates was immunoprecipitated using anti-FLAG, then immunoprecipitates were analyzed for ubiquitination of ATG101 by Western blotting using anti-HA. * non-specific bands. (b) The transfected cells same in (a) were performed vice versa; which were immunoprecipitated using anti-HA, then the immunoprecipitated complexes of HA-Ub were analyzed by Western blotting using anti-ATG101. * non-specific bands.

Figure S3. (a) Schematics for generating an ATG101 C-terminal domain deletion mutant (ATG101ΔC). (b) Physical interactions of ATG101ΔC with other ATG proteins. HEK293T cells were co-transfected with FLAG-ATG101 or ΔC and either HA-ATG13 or HA-ATG14 respectively and incubated for 24–48 h and then cells were incubated in amino-acid deprived medium (−AA) for 2 h prior to harvesting. FLAG-ATG101 was immunoprecipitated with anti-FLAG, then the immunoprecipitated complexes were analyzed for interaction between ATG101 and HA-ATG13/ATG14 by Western blotting using anti-ATG13/ATG14.

Figure S4. ATG101 Single lysine mutant in C-terminal region is not sufficient for altering autophagy activity. (a) Western blot analysis of ATG101 lysine mutants. ATG101 KO MIA PaCa-2 cells were plated overnight and transfected with FLAG-Vector (Vec), ATG101 WT(WT), ATG101ΔC (ΔC), ATG101 K105R(K105R), and ATG101 K213R (K213R). Cells were then treated with CHX (10 μg/ml) and lysed at the time indicated. Cell lysates were immunoblotted against ATG101. ATG101 levels were normalized to β-actin, which served as the gel loading control. Western blot band intensities were quantified by Image J. Quantification data is presented on the graph. Error bars indicate as mean ± SEM of three independent experiments. * $P < 0.05$; ** $P < 0.01$. (b) Comparison of ubiquitination levels among ATG101 lysine mutants. HA-ubiquitin (HA-Ub) and FLAG-ATG101 WT, ΔC, K105R (105), or K213R (213) plasmids were co-transfected into ATG101 KO HEK293T cells. Cell lysates were immunoprecipitated with anti-FLAG antibody and ATG101 ubiquitination determined by immunoblotting against HA and FLAG. (c) Autophagosome formation in cells expressing an ATG101 lysine mutant. ATG101 KO MIA PaCa-2 cells stably expressing GFP-LC3 were plated overnight and transfected with FLAG vector (Vec) and ATG101 WT or K213R for 48 h. Cells were incubated in amino-acid deprived medium (−AA) for 4 h prior to image acquisition by confocal microscopy. Images were acquired using a confocal fluorescence microscope. Imaging methods, quantification, and statistical analysis are as described in Figure 4c. * $P < 0.05$.

Figure S5. Ubiquitination levels of ATG101 in starvation condition were similar to the those in nutrient basal condition. shCTL or shHUWE1 HEK293T cells were co-transfected with HA-Ub and FLAG-ATG101. FLAG-ATG101 was immunoprecipitated with anti-FLAG, then the immunoprecipitated complexes were analyzed for ATG101 ubiquitination by Western blotting using anti-HA. * non-specific bands.

Figure S6. (a) Confirmation of ATG101 knockdown for figure 4c by Western blotting analysis. (b) Confirmation of ATG101 and/or WIPI2 knockdown for figure 4d by Western blotting analysis. Corresponding cells (figure 4c,d) were transfected either ATG101 and/or WIPI2 siRNA for 48 h, followed by incubation in rapamycin (0.5 μM) (a) amino acid-deprived medium (−AA) (b) for 4 h. Cell lysates were collected and immunoblotted for ATG101, WIPI2 and β-actin (gel loading control). (c) Additional knockdown of ATG101 and/or WIPI2 reduced colony formation. shCTL or shHUWE1 MIA PaCa-2 cells were plated with 500 cells/well and transfected with WIPI2 and/or ATG101 siRNA for 24 h. Then, culture medium was changed with fresh complete medium and incubated further for 4 days. Colonies were fixed and stained with Crystal violet (0.5%).
