# Supplementary material for: ATG101 Degradation by HUWE1-Mediated Ubiquitination Impairs Autophagy and Reduces Survival in Cancer Cells
Source: Int J Mol Sci. 2021 Aug 25;22(17):9182. doi: 10.3390/ijms22179182 (PMC8430637; doi:10.3390/ijms22179182)
Supplement: Supplementary file 1 [file ijms-22-09182-s001.zip › IJMS1299763_Supplementary materials_Revised.pptx]

## Slide 1
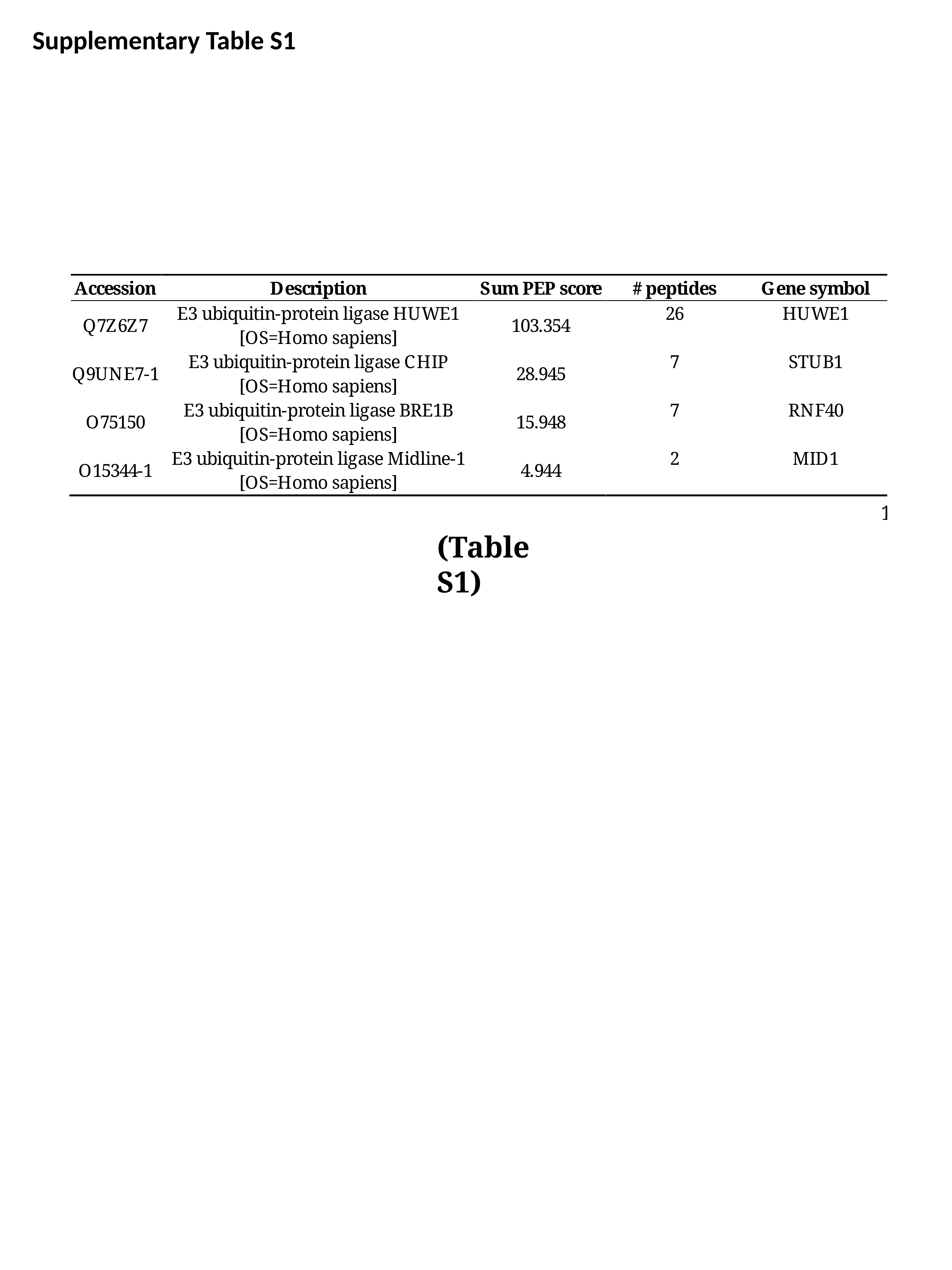

Supplementary Table S1
(Table S1)

## Slide 2
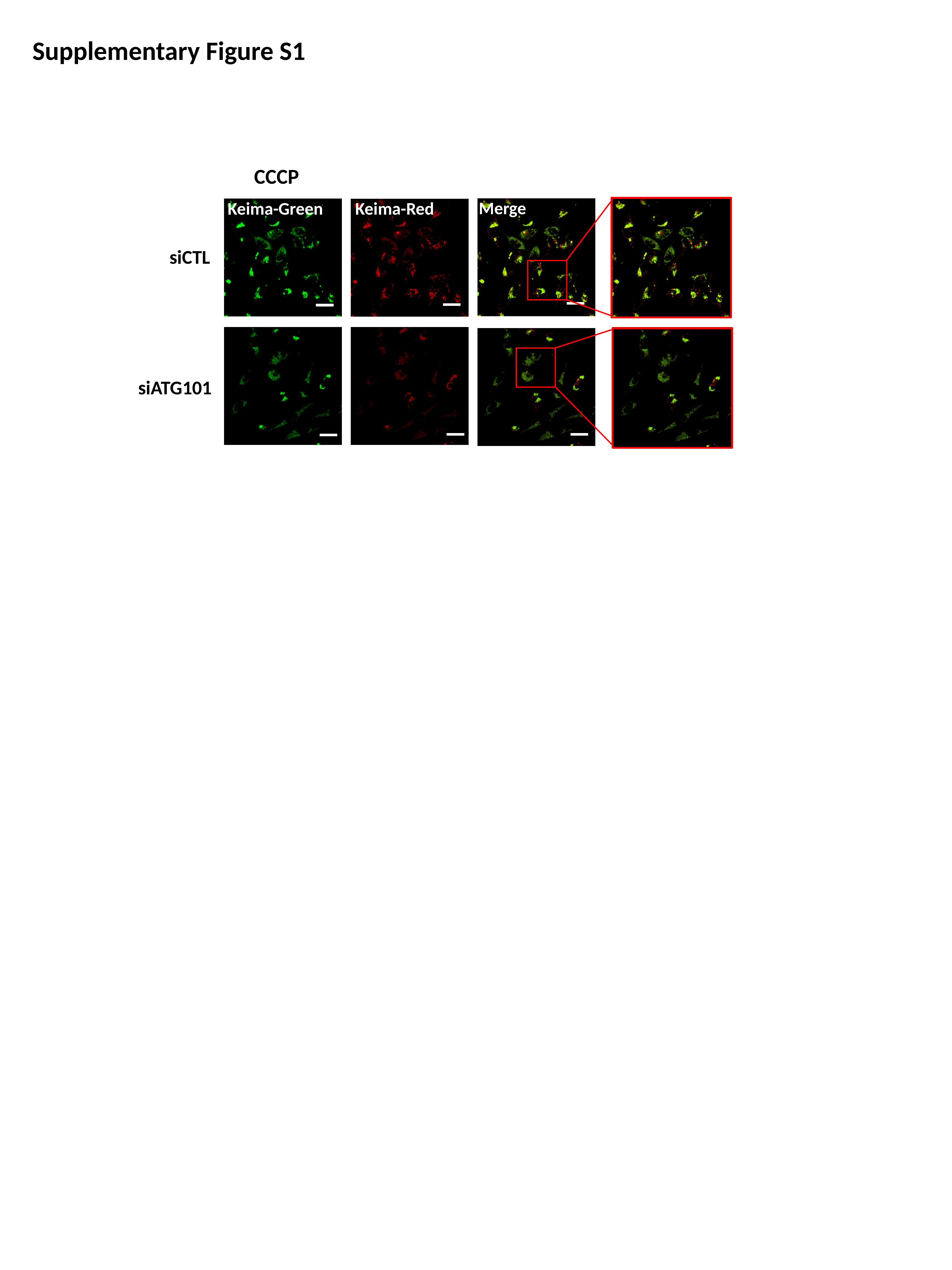

Supplementary Figure S1
CCCP
Merge
Keima-Green
Keima-Red
siCTL
siATG101

## Slide 3
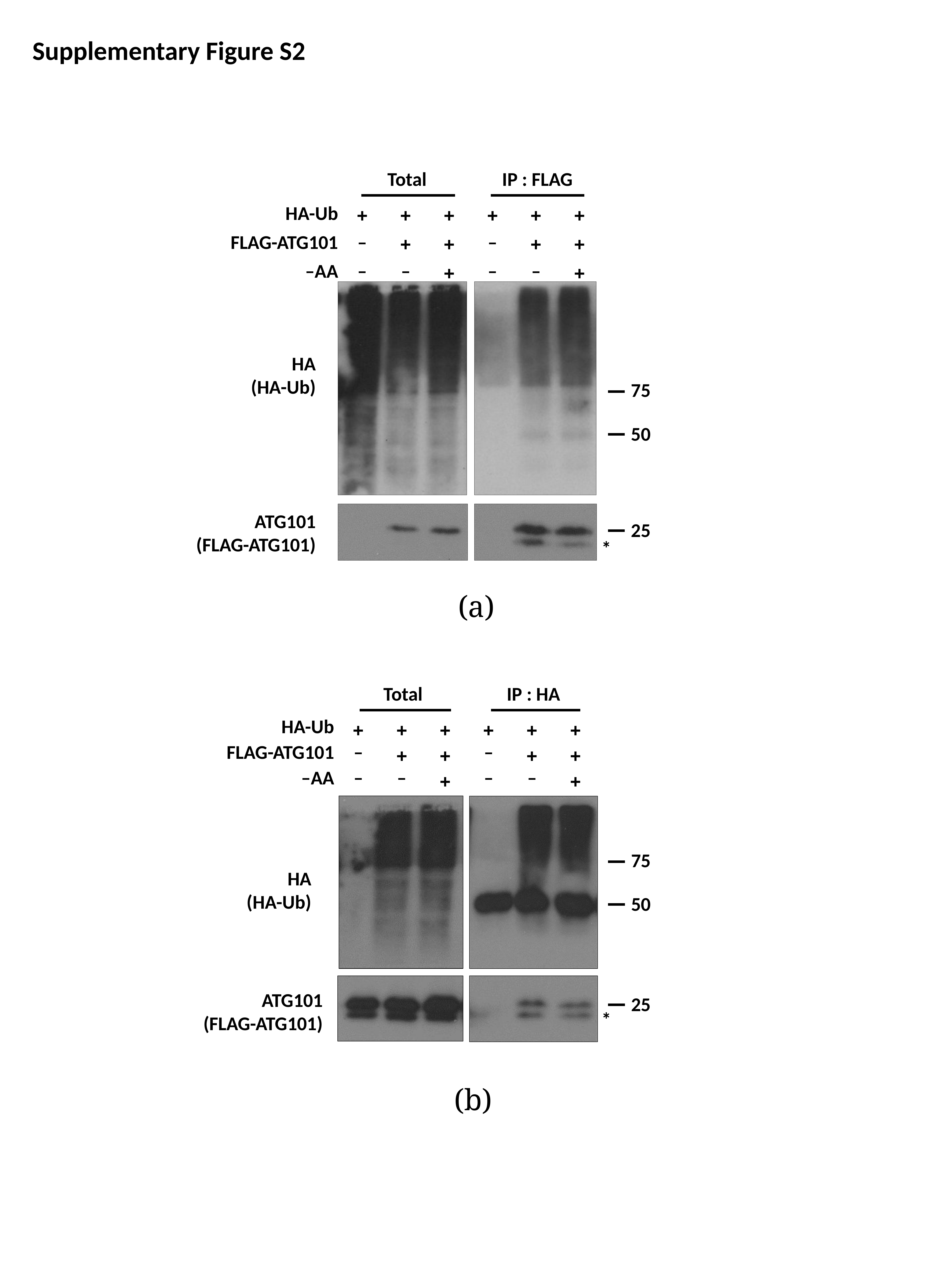

Supplementary Figure S2
Total
IP : FLAG
| HA-Ub | + | + | + | + | + | + |
| --- | --- | --- | --- | --- | --- | --- |
| FLAG-ATG101 | – | + | + | – | + | + |
| –AA | – | – | + | – | – | + |
HA
(HA-Ub)
75
50
*
ATG101
(FLAG-ATG101)
25
*
(a)
Total
IP : HA
| HA-Ub | + | + | + | + | + | + |
| --- | --- | --- | --- | --- | --- | --- |
| FLAG-ATG101 | – | + | + | – | + | + |
| –AA | – | – | + | – | – | + |
75
HA
(HA-Ub)
50
*
ATG101
(FLAG-ATG101)
25
*
(b)

## Slide 4
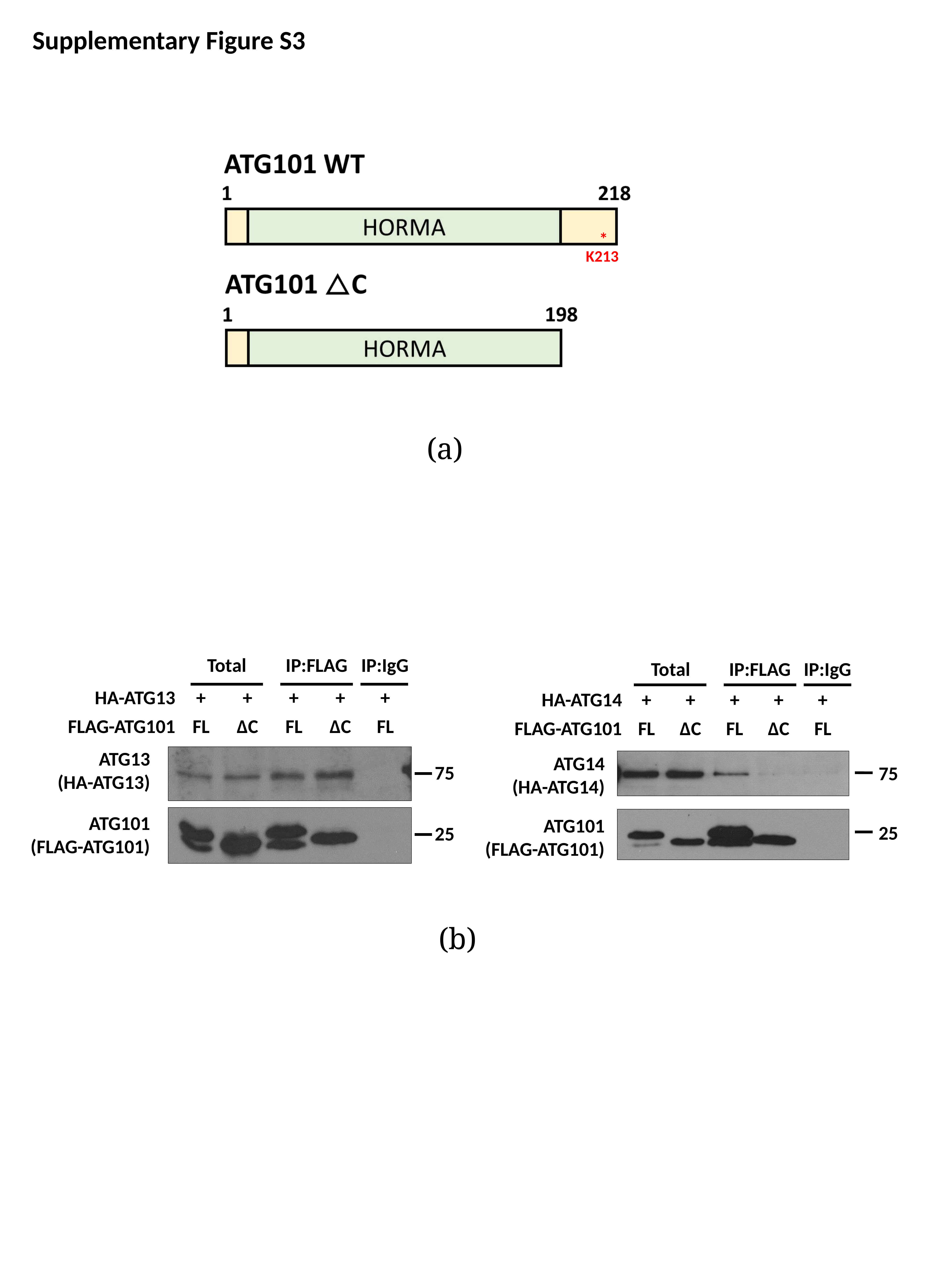

Supplementary Figure S3
*
K213
(a)
Total
IP:FLAG
IP:IgG
Total
IP:FLAG
IP:IgG
| HA-ATG13 | + | + | + | + | + |
| --- | --- | --- | --- | --- | --- |
| FLAG-ATG101 | FL | ΔC | FL | ΔC | FL |
| HA-ATG14 | + | + | + | + | + |
| --- | --- | --- | --- | --- | --- |
| FLAG-ATG101 | FL | ΔC | FL | ΔC | FL |
ATG13
(HA-ATG13)
ATG14
(HA-ATG14)
75
75
ATG101
(FLAG-ATG101)
ATG101
(FLAG-ATG101)
25
25
(b)

## Slide 5
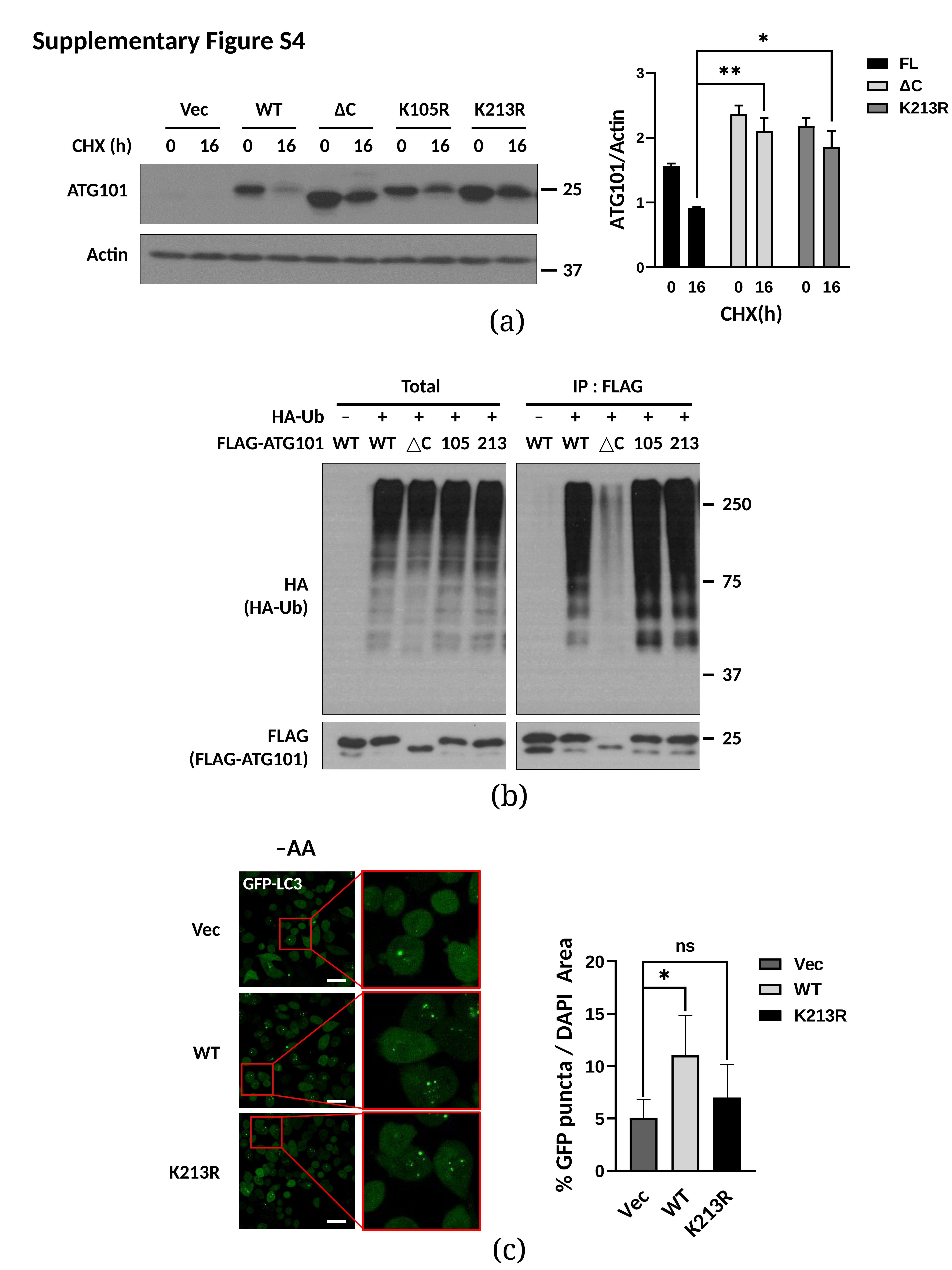

Supplementary Figure S4
| Vec |
| --- |
| WT |
| --- |
| ΔC |
| --- |
| K105R |
| --- |
| K213R |
| --- |
| CHX (h) | 0 | 16 | 0 | 16 | 0 | 16 | 0 | 16 | 0 | 16 |
| --- | --- | --- | --- | --- | --- | --- | --- | --- | --- | --- |
25
ATG101
Actin
37
(a)
| Total |
| --- |
| IP : FLAG |
| --- |
| HA-Ub | – | + | + | + | + | | – | + | + | + | + |
| --- | --- | --- | --- | --- | --- | --- | --- | --- | --- | --- | --- |
| FLAG-ATG101 | WT | WT | △C | 105 | 213 | | WT | WT | △C | 105 | 213 |
250
75
HA
(HA-Ub)
37
FLAG
(FLAG-ATG101)
25
(b)
–AA
GFP-LC3
Vec
WT
K213R
(c)

## Slide 6
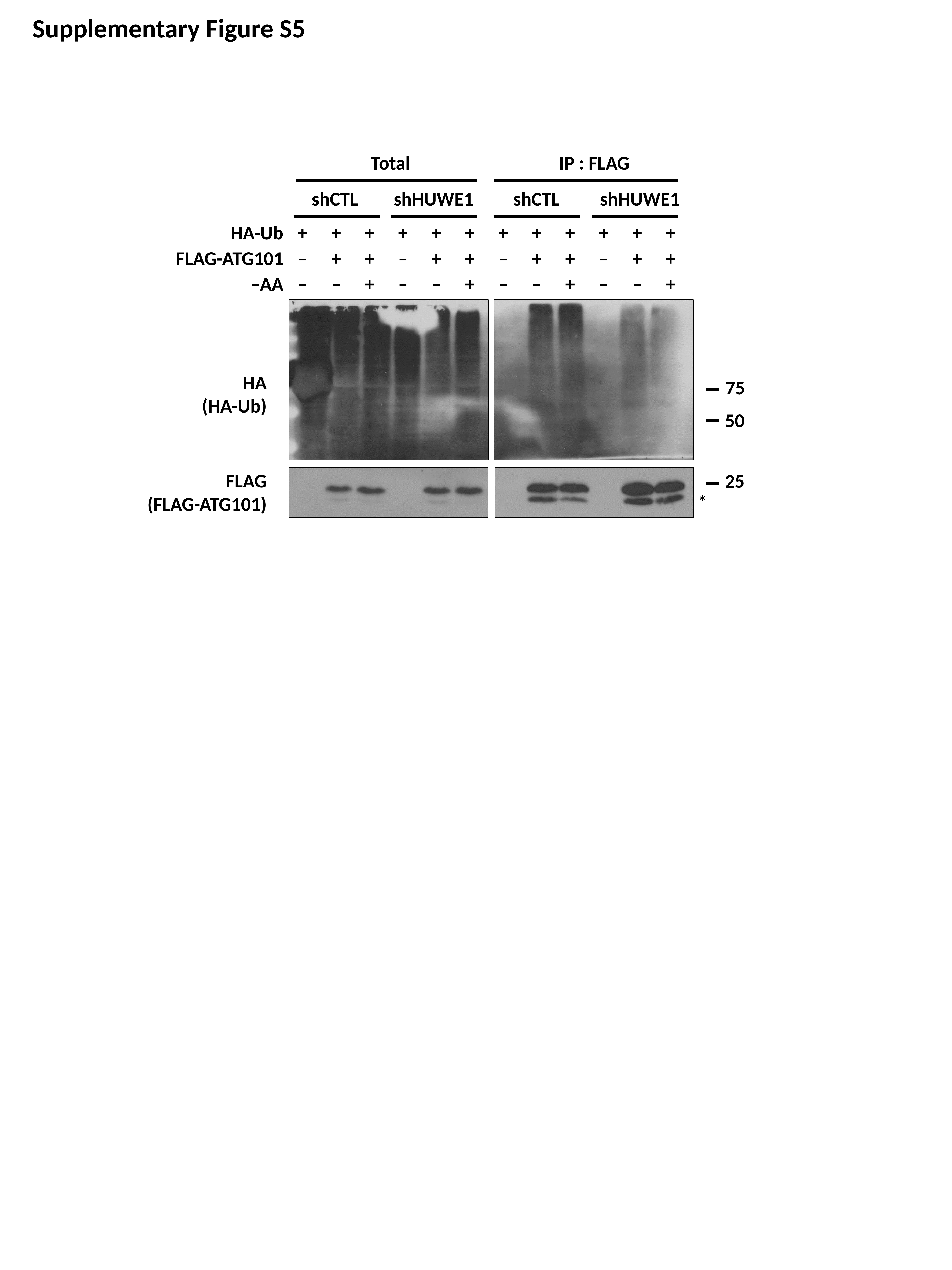

Supplementary Figure S5
IP : FLAG
Total
shHUWE1
shHUWE1
shCTL
shCTL
| HA-Ub | + | + | + | + | + | + | + | + | + | + | + | + |
| --- | --- | --- | --- | --- | --- | --- | --- | --- | --- | --- | --- | --- |
| FLAG-ATG101 | – | + | + | – | + | + | – | + | + | – | + | + |
| –AA | – | – | + | – | – | + | – | – | + | – | – | + |
HA
(HA-Ub)
75
50
FLAG
(FLAG-ATG101)
25
*

## Slide 7
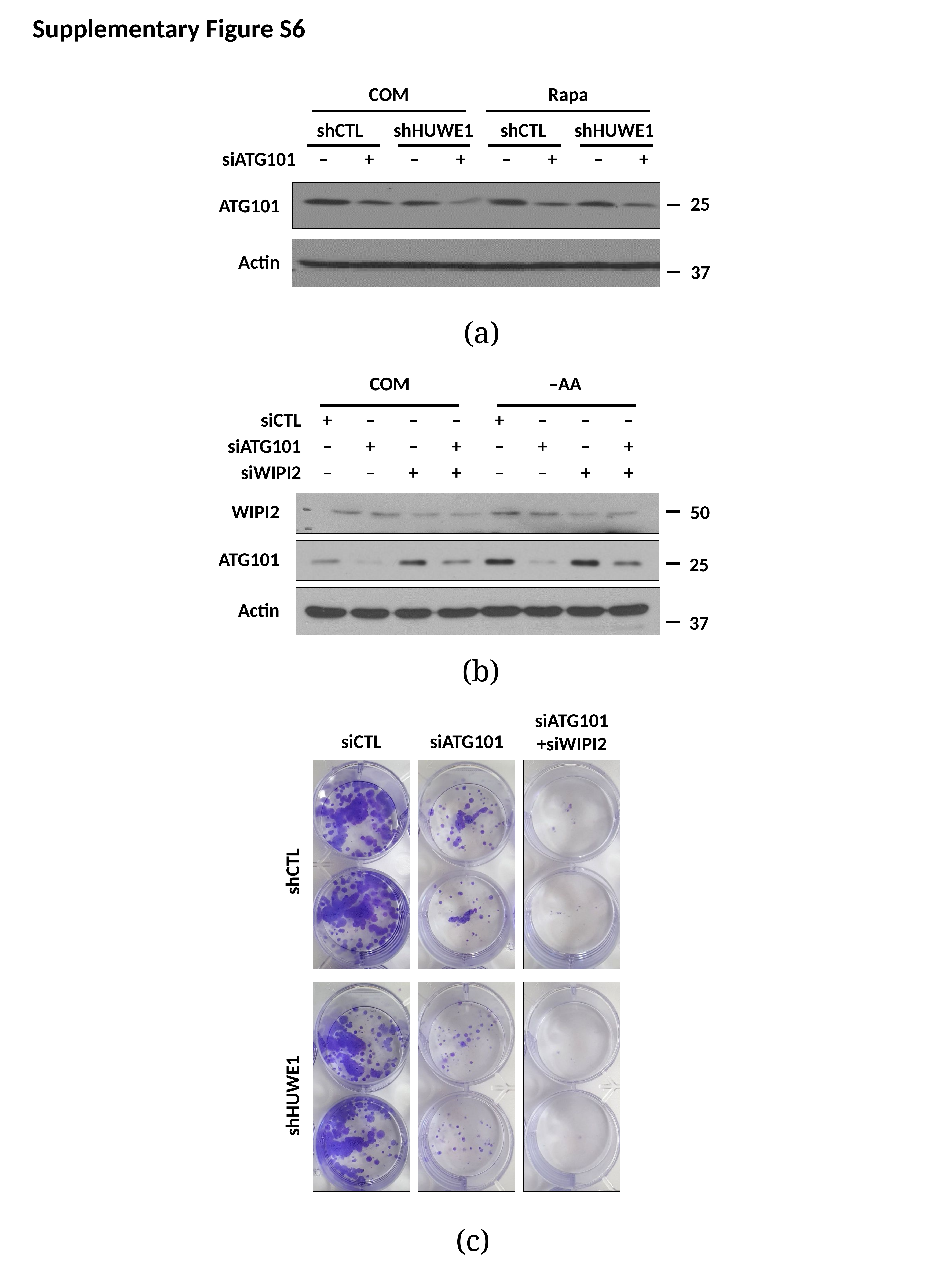

Supplementary Figure S6
COM
Rapa
shCTL
shHUWE1
shCTL
shHUWE1
| siATG101 | – | + | – | + | – | + | – | + |
| --- | --- | --- | --- | --- | --- | --- | --- | --- |
25
ATG101
Actin
37
(a)
COM
–AA
| siCTL | + | – | – | – | + | – | – | – |
| --- | --- | --- | --- | --- | --- | --- | --- | --- |
| siATG101 | – | + | – | + | – | + | – | + |
| siWIPI2 | – | – | + | + | – | – | + | + |
50
WIPI2
ATG101
25
Actin
37
(b)
siATG101
+siWIPI2
siATG101
siCTL
shCTL
shHUWE1
(c)
