# Supplementary material for: ATG101 Degradation by HUWE1-Mediated Ubiquitination Impairs Autophagy and Reduces Survival in Cancer Cells
Source: Int J Mol Sci. 2021 Aug 25;22(17):9182. doi: 10.3390/ijms22179182 (PMC8430637; doi:10.3390/ijms22179182)
Supplement: Supplementary file 1 [file ijms-22-09182-s001.zip › Supplementary Figure S3.pptx]

## Slide 1
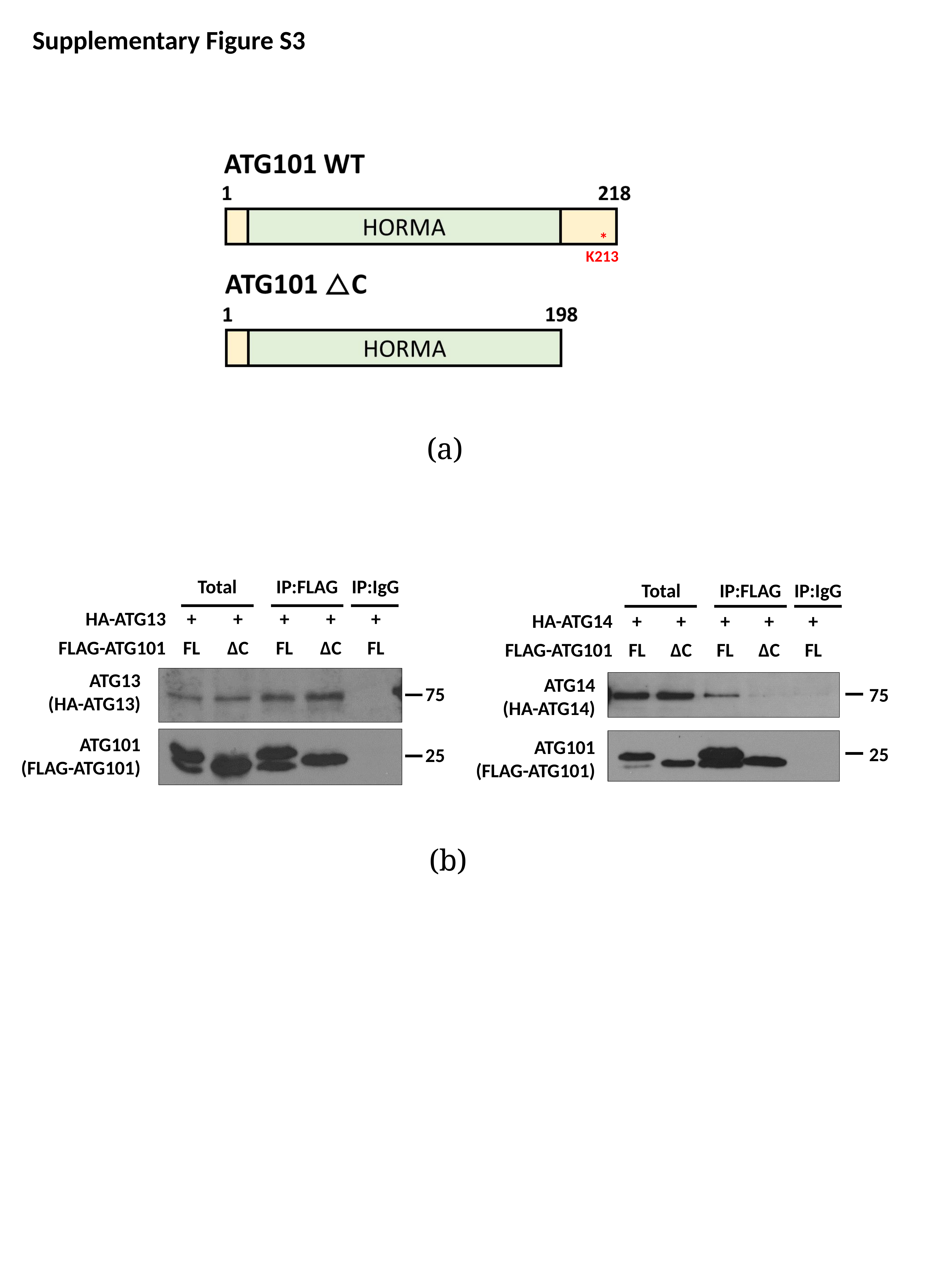

Supplementary Figure S3
*
K213
(a)
Total
IP:FLAG
IP:IgG
Total
IP:FLAG
IP:IgG
| HA-ATG13 | + | + | + | + | + |
| --- | --- | --- | --- | --- | --- |
| FLAG-ATG101 | FL | ΔC | FL | ΔC | FL |
| HA-ATG14 | + | + | + | + | + |
| --- | --- | --- | --- | --- | --- |
| FLAG-ATG101 | FL | ΔC | FL | ΔC | FL |
ATG13
(HA-ATG13)
ATG14
(HA-ATG14)
75
75
ATG101
(FLAG-ATG101)
ATG101
(FLAG-ATG101)
25
25
(b)
