# Supplementary material for: ATG101 Degradation by HUWE1-Mediated Ubiquitination Impairs Autophagy and Reduces Survival in Cancer Cells
Source: Int J Mol Sci. 2021 Aug 25;22(17):9182. doi: 10.3390/ijms22179182 (PMC8430637; doi:10.3390/ijms22179182)
Supplement: Supplementary file 1 [file ijms-22-09182-s001.zip › Supplementary Figure S4.pptx]

## Slide 1
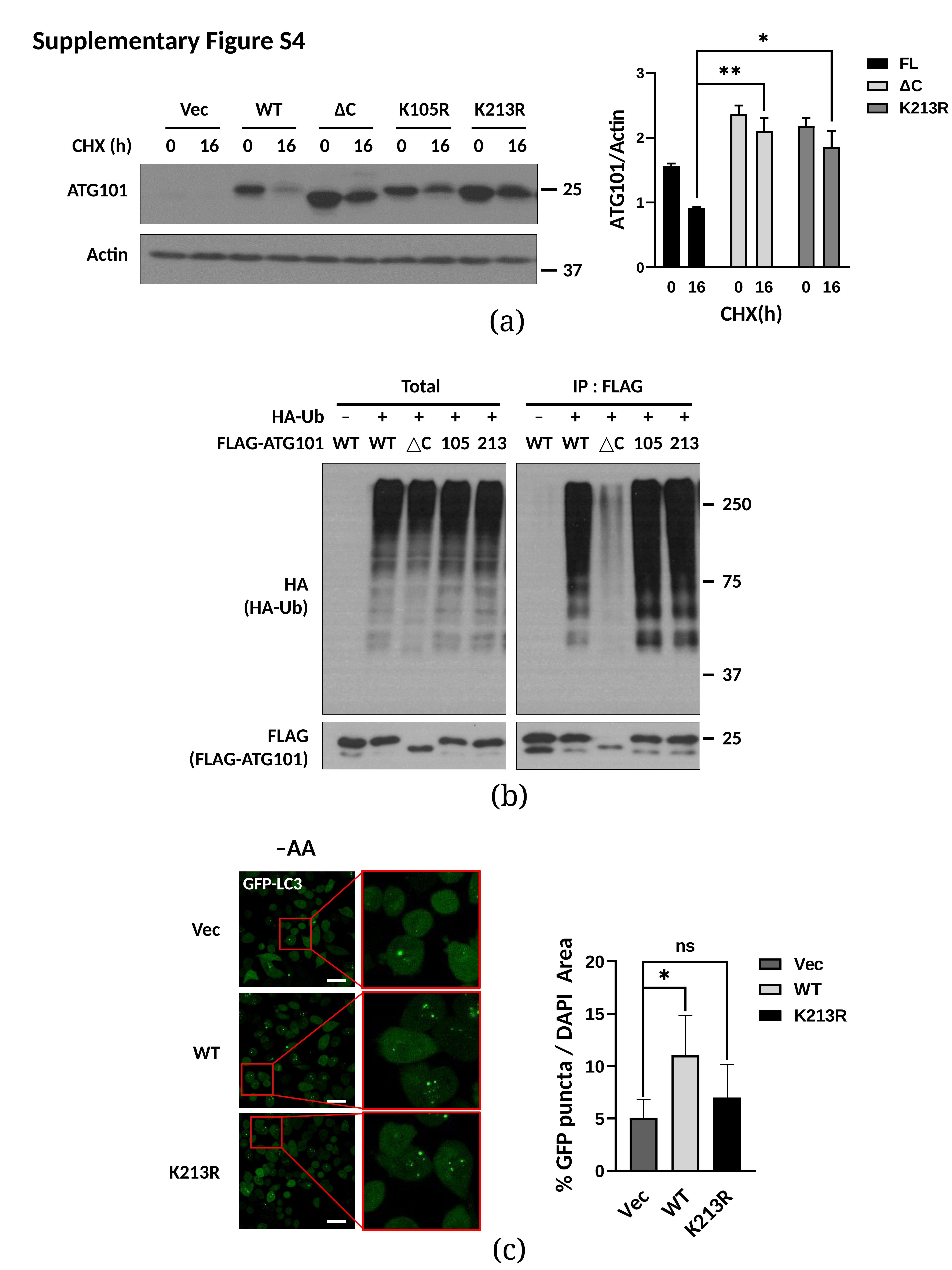

Supplementary Figure S4
| Vec |
| --- |
| WT |
| --- |
| ΔC |
| --- |
| K105R |
| --- |
| K213R |
| --- |
| CHX (h) | 0 | 16 | 0 | 16 | 0 | 16 | 0 | 16 | 0 | 16 |
| --- | --- | --- | --- | --- | --- | --- | --- | --- | --- | --- |
25
ATG101
Actin
37
(a)
| Total |
| --- |
| IP : FLAG |
| --- |
| HA-Ub | – | + | + | + | + | | – | + | + | + | + |
| --- | --- | --- | --- | --- | --- | --- | --- | --- | --- | --- | --- |
| FLAG-ATG101 | WT | WT | △C | 105 | 213 | | WT | WT | △C | 105 | 213 |
250
75
HA
(HA-Ub)
37
FLAG
(FLAG-ATG101)
25
(b)
–AA
GFP-LC3
Vec
WT
K213R
(c)
