# Supplementary material for: ATG101 Degradation by HUWE1-Mediated Ubiquitination Impairs Autophagy and Reduces Survival in Cancer Cells
Source: Int J Mol Sci. 2021 Aug 25;22(17):9182. doi: 10.3390/ijms22179182 (PMC8430637; doi:10.3390/ijms22179182)
Supplement: Supplementary file 1 [file ijms-22-09182-s001.zip › Supplementary Figure S5.pptx]

## Slide 1
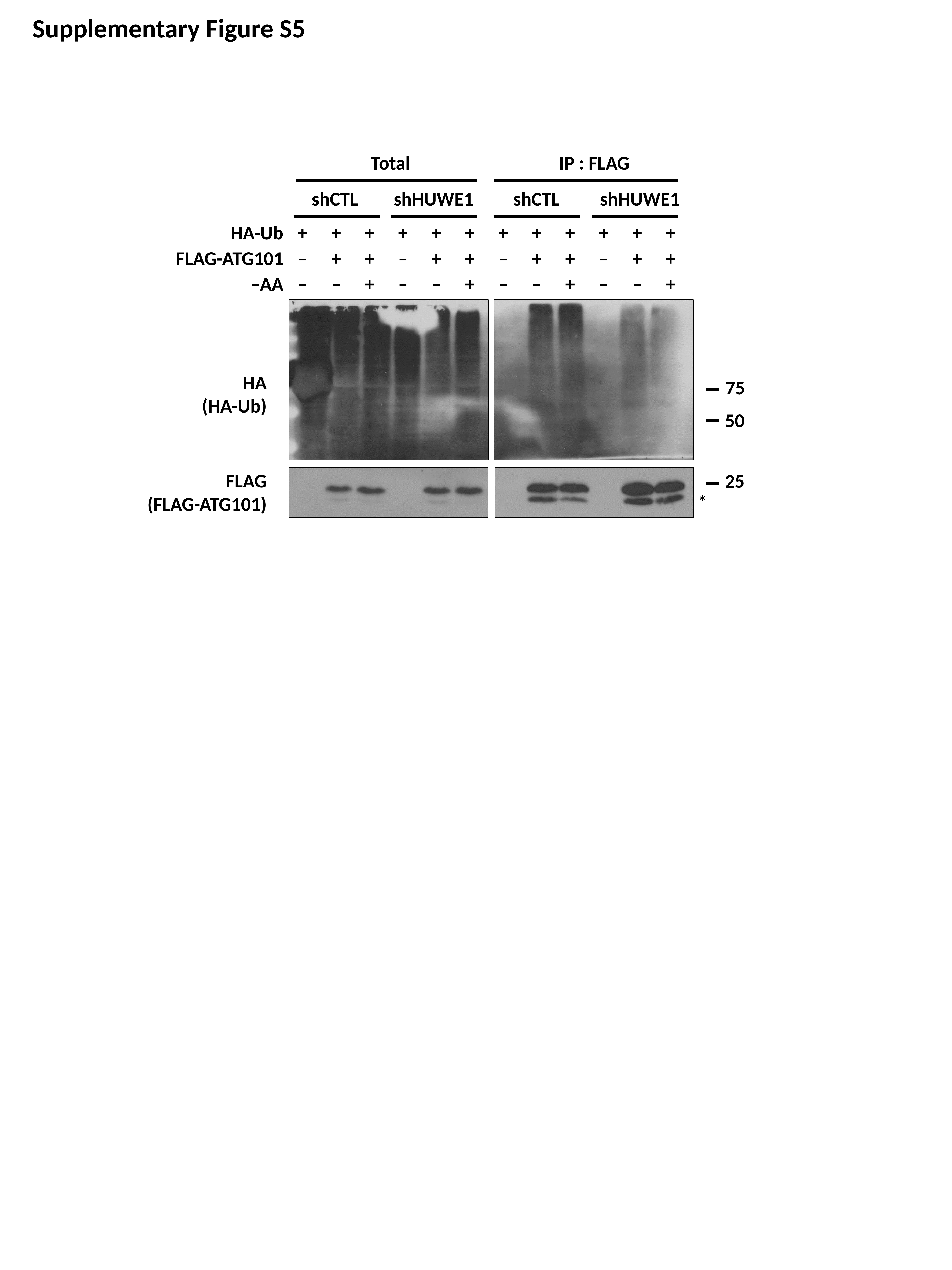

Supplementary Figure S5
IP : FLAG
Total
shHUWE1
shHUWE1
shCTL
shCTL
| HA-Ub | + | + | + | + | + | + | + | + | + | + | + | + |
| --- | --- | --- | --- | --- | --- | --- | --- | --- | --- | --- | --- | --- |
| FLAG-ATG101 | – | + | + | – | + | + | – | + | + | – | + | + |
| –AA | – | – | + | – | – | + | – | – | + | – | – | + |
HA
(HA-Ub)
75
50
FLAG
(FLAG-ATG101)
25
*
