# Supplementary material for: ATG101 Degradation by HUWE1-Mediated Ubiquitination Impairs Autophagy and Reduces Survival in Cancer Cells
Source: Int J Mol Sci. 2021 Aug 25;22(17):9182. doi: 10.3390/ijms22179182 (PMC8430637; doi:10.3390/ijms22179182)
Supplement: Supplementary file 1 [file ijms-22-09182-s001.zip › Supplementary Figure S6.pptx]

## Slide 1
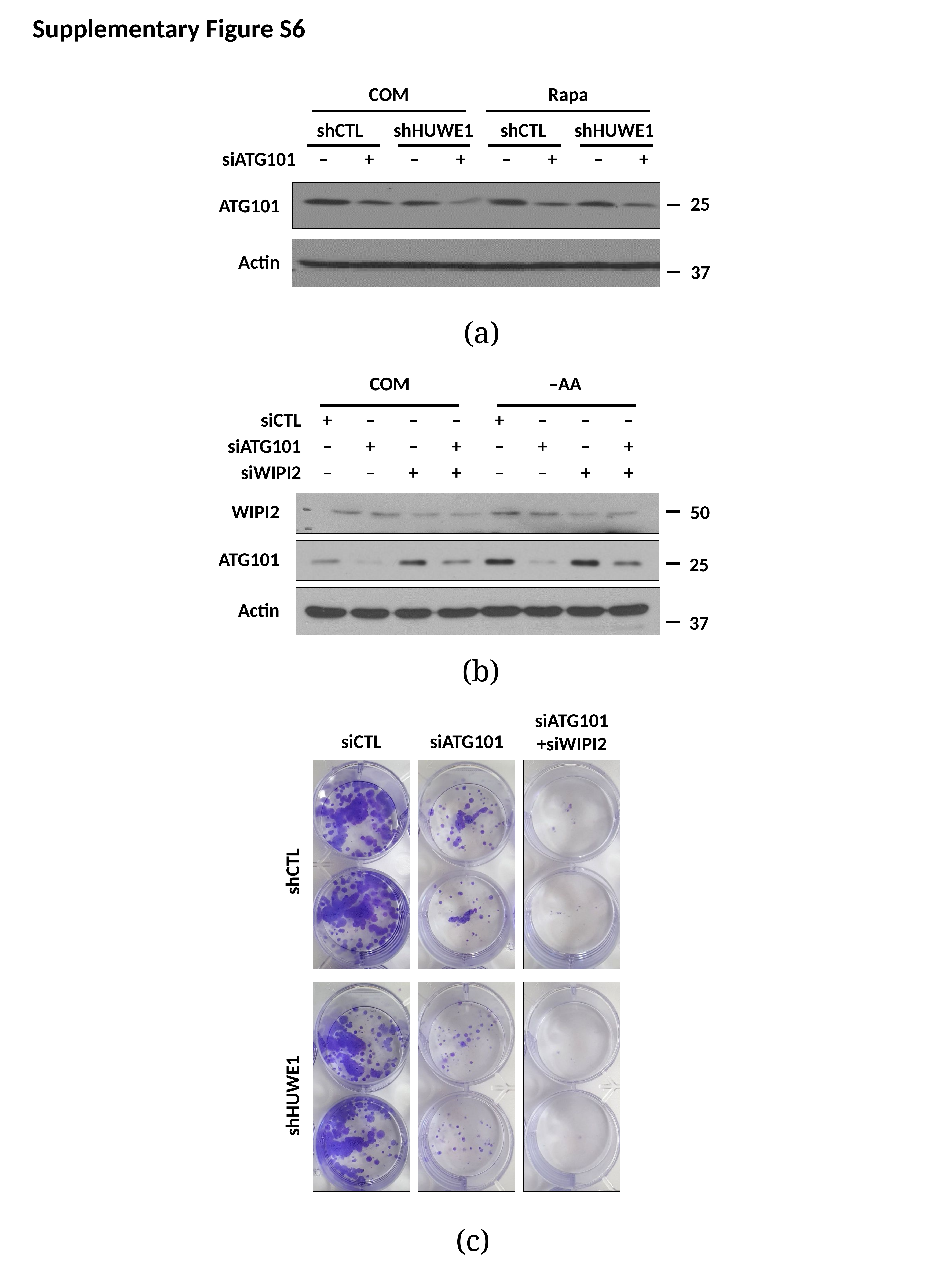

Supplementary Figure S6
COM
Rapa
shCTL
shHUWE1
shCTL
shHUWE1
| siATG101 | – | + | – | + | – | + | – | + |
| --- | --- | --- | --- | --- | --- | --- | --- | --- |
25
ATG101
Actin
37
(a)
COM
–AA
| siCTL | + | – | – | – | + | – | – | – |
| --- | --- | --- | --- | --- | --- | --- | --- | --- |
| siATG101 | – | + | – | + | – | + | – | + |
| siWIPI2 | – | – | + | + | – | – | + | + |
50
WIPI2
ATG101
25
Actin
37
(b)
siATG101
+siWIPI2
siATG101
siCTL
shCTL
shHUWE1
(c)
