# Supplementary material for: ATG101 Degradation by HUWE1-Mediated Ubiquitination Impairs Autophagy and Reduces Survival in Cancer Cells
Source: Int J Mol Sci. 2021 Aug 25;22(17):9182. doi: 10.3390/ijms22179182 (PMC8430637; doi:10.3390/ijms22179182)
Supplement: Supplementary file 1 [file ijms-22-09182-s001.zip › Supplementary Table S1.pdf]

Supplementary Table S1

Accession	Description	Sum PEP score	# peptides	Gene symbol
Q7Z6Z7	E3 ubiquitin-protein ligase HUWE1 [OS=Homo sapiens]	103.354	26	HUWE1
Q9UNE7-1	E3 ubiquitin-protein ligase CHIP [OS=Homo sapiens]	28.945	7	STUB1
O75150	E3 ubiquitin-protein ligase BRE1B [OS=Homo sapiens]	15.948	7	RNF40
O15344-1	E3 ubiquitin-protein ligase Midline-1 [OS=Homo sapiens]	4.944	2	MID1

(Table S1)
