# Supplementary figures and images for: ATG101 Degradation by HUWE1-Mediated Ubiquitination Impairs Autophagy and Reduces Survival in Cancer Cells
Source: Int J Mol Sci. 2021 Aug 25;22(17):9182. doi: 10.3390/ijms22179182 (PMC8430637; doi:10.3390/ijms22179182)

Supplementary Figure S1

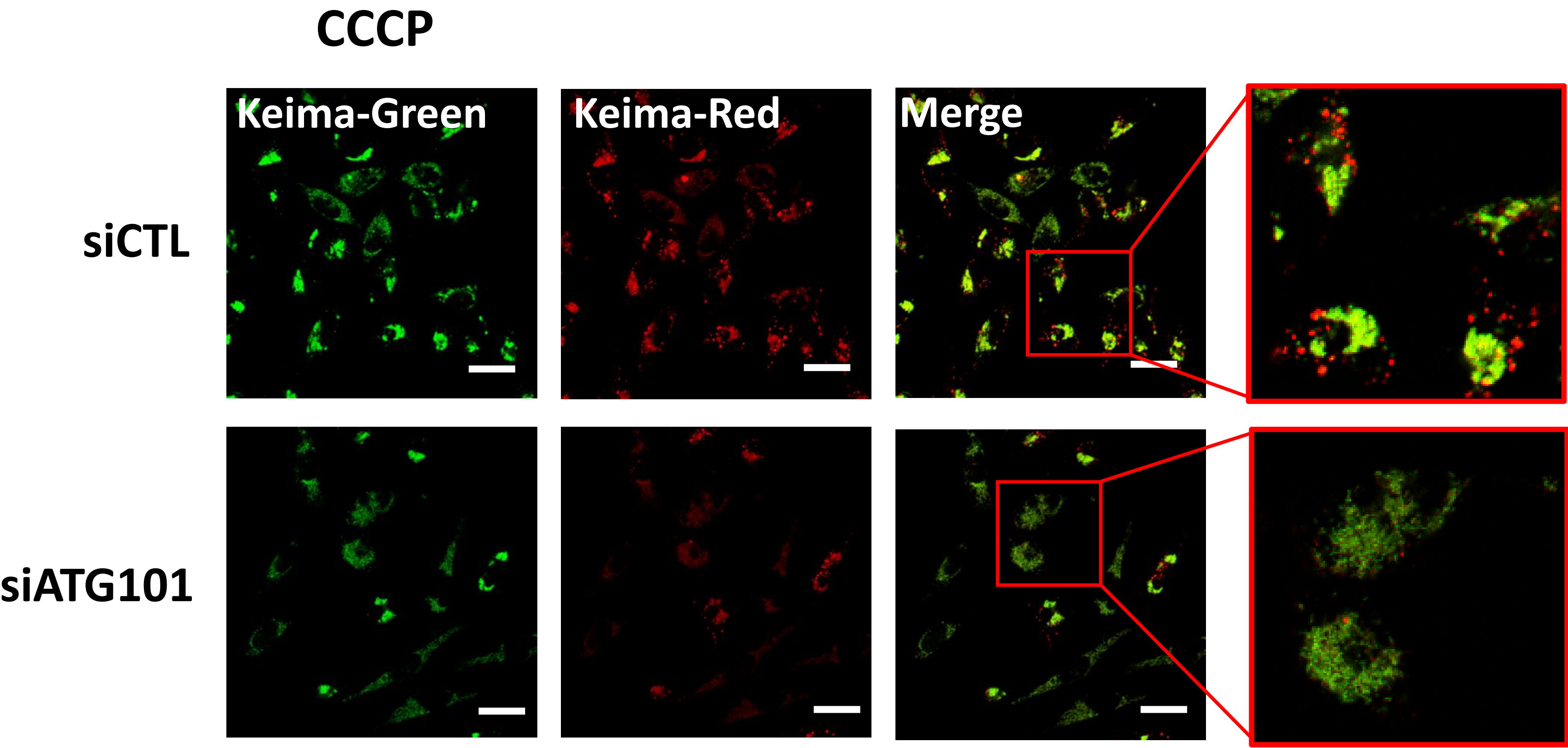

(a)

Supplement: Supplementary file 1 [file ijms-22-09182-s001.zip › Supplementary Figure S1.pdf]

## Slide 1
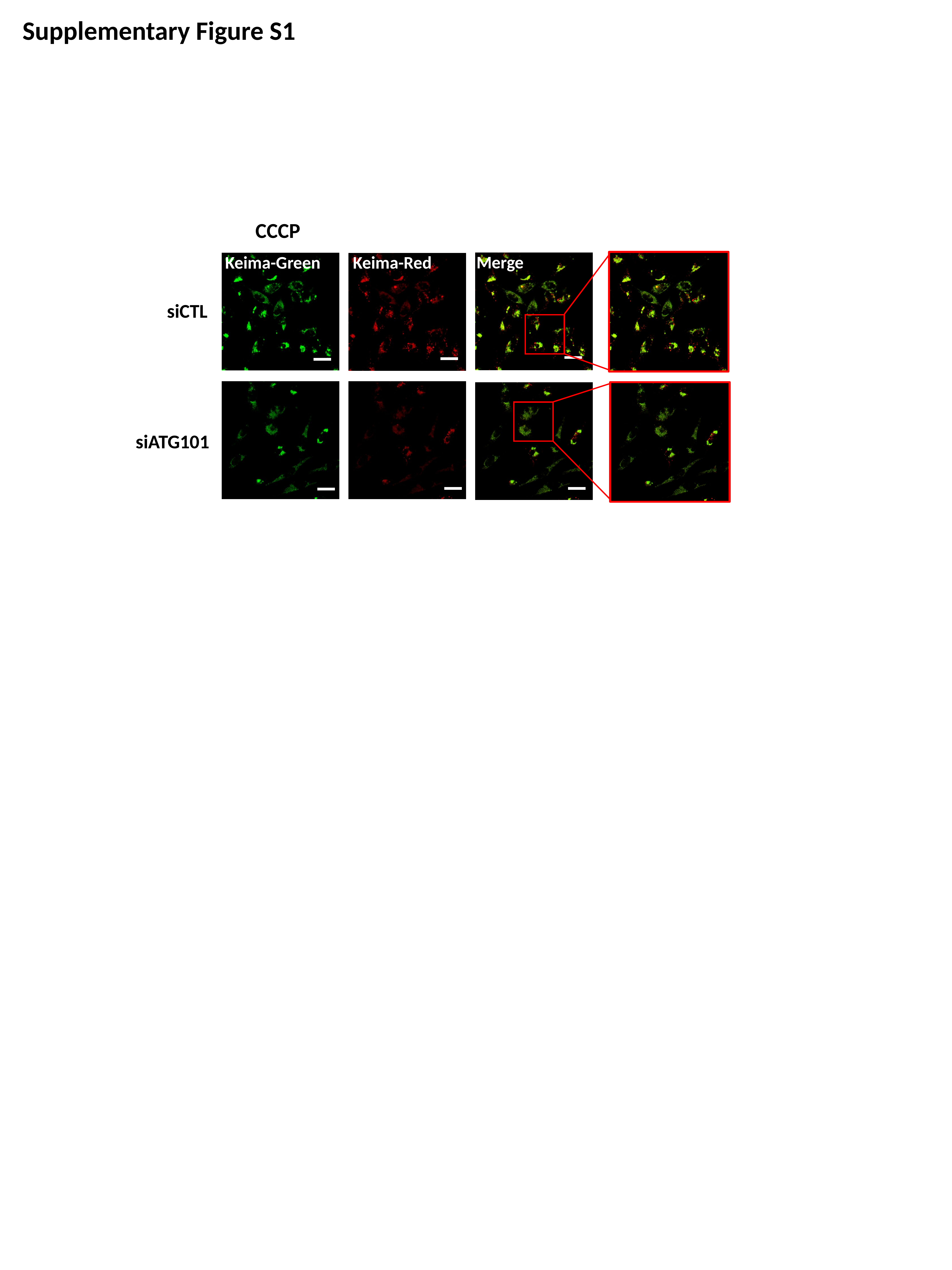

Supplementary Figure S1
CCCP
Merge
Keima-Green
Keima-Red
siCTL
siATG101

Supplement: Supplementary file 1 [file ijms-22-09182-s001.zip › Supplementary Figure S1.pptx]

Supplementary Figure S2

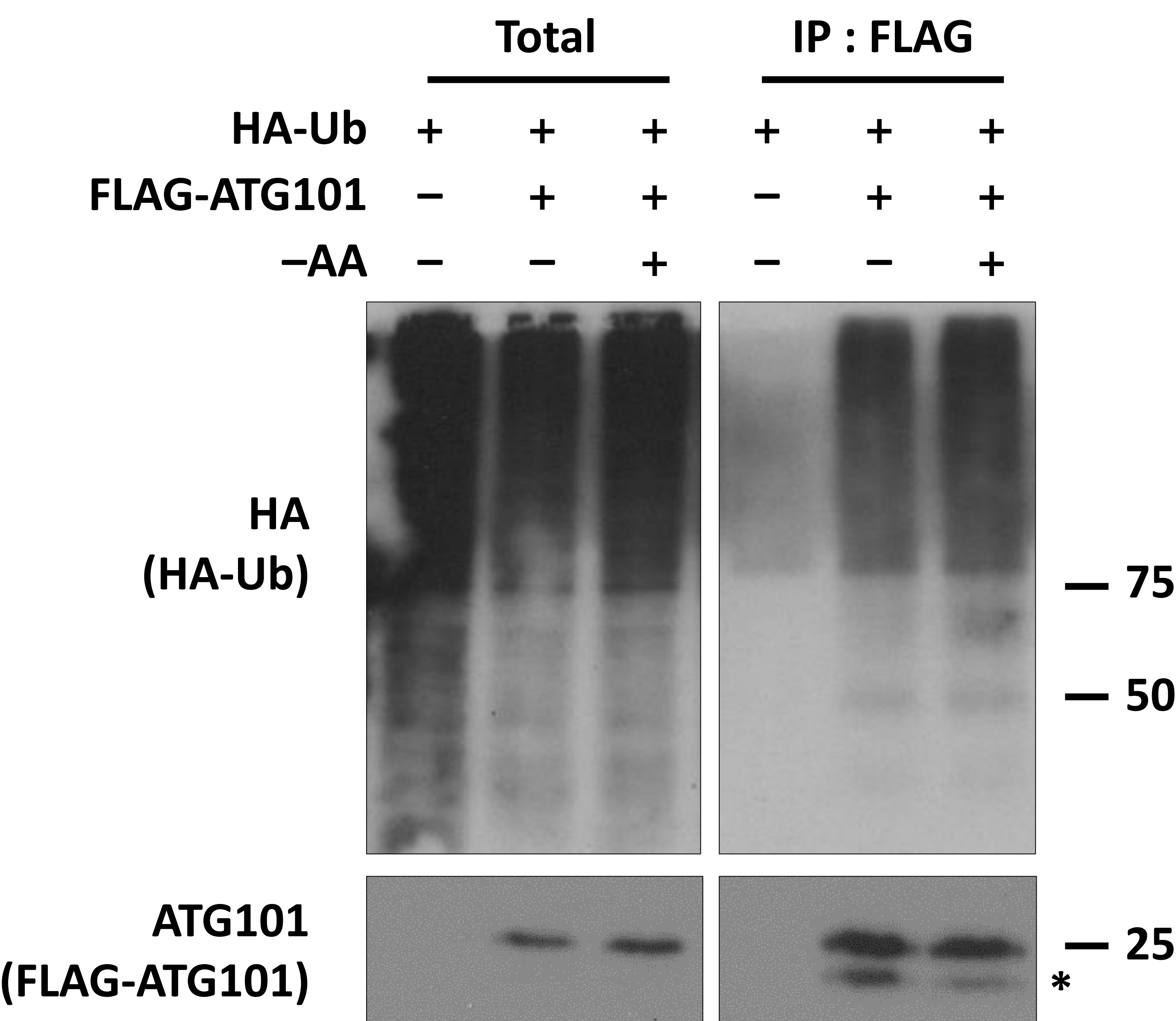

(a)

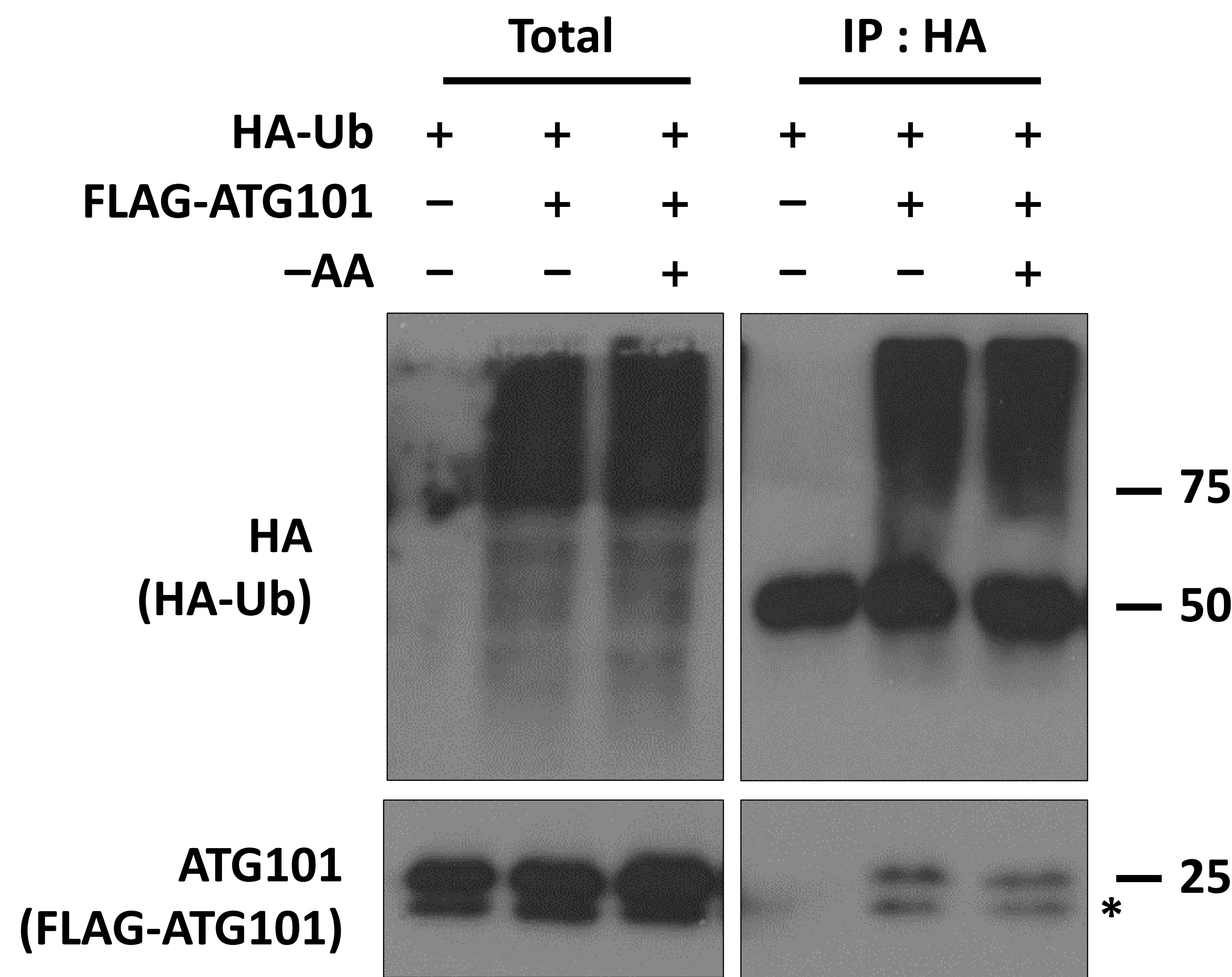

(b)

Supplement: Supplementary file 1 [file ijms-22-09182-s001.zip › Supplementary Figure S2.pdf]

Supplementary Figure S3

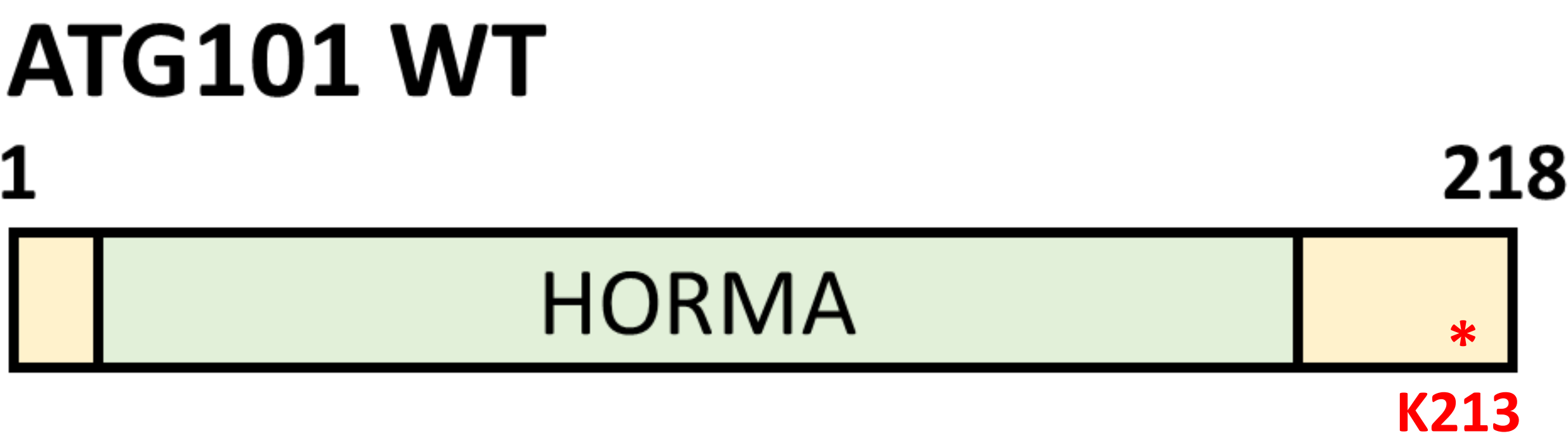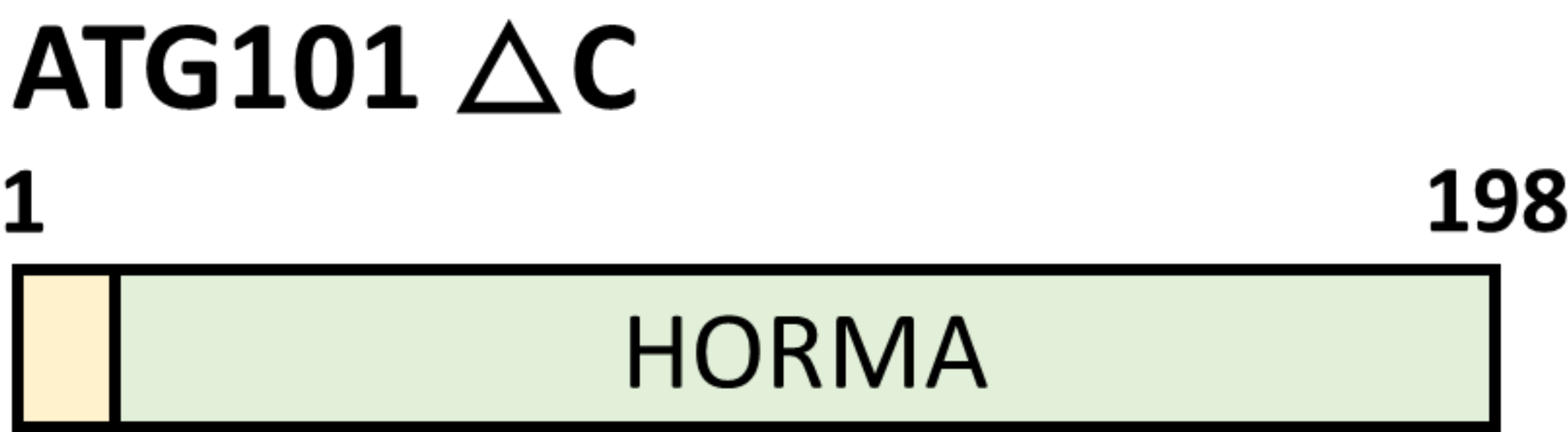

(a)

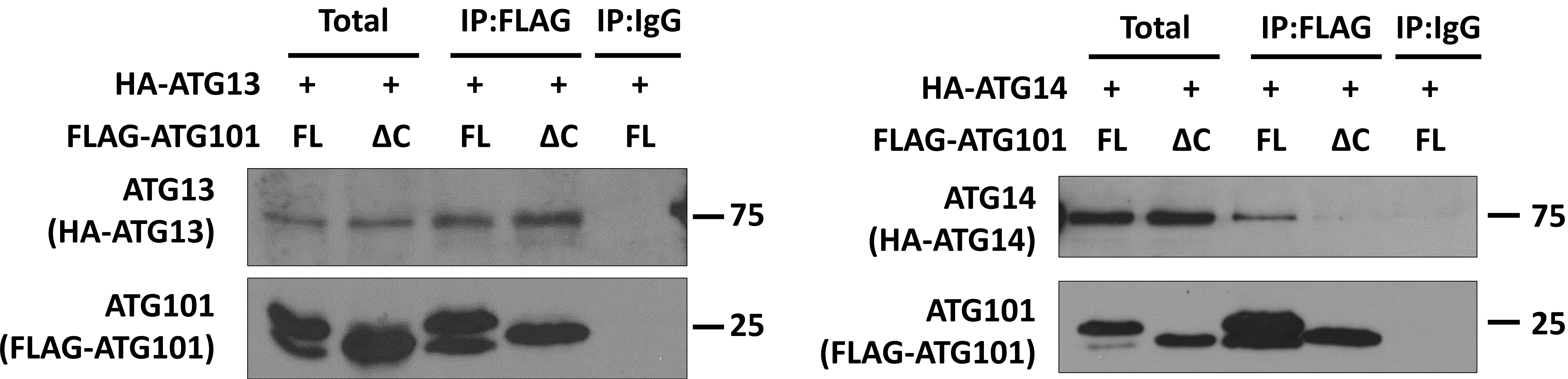

(b)

Supplement: Supplementary file 1 [file ijms-22-09182-s001.zip › Supplementary Figure S3.pdf]

Supplementary Figure S4

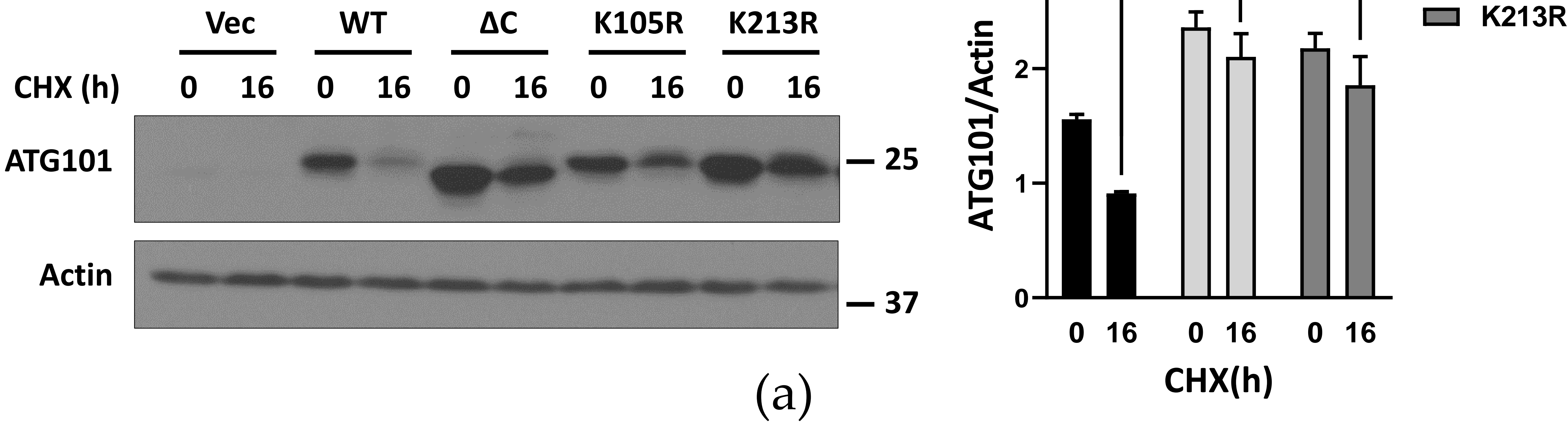

(a)

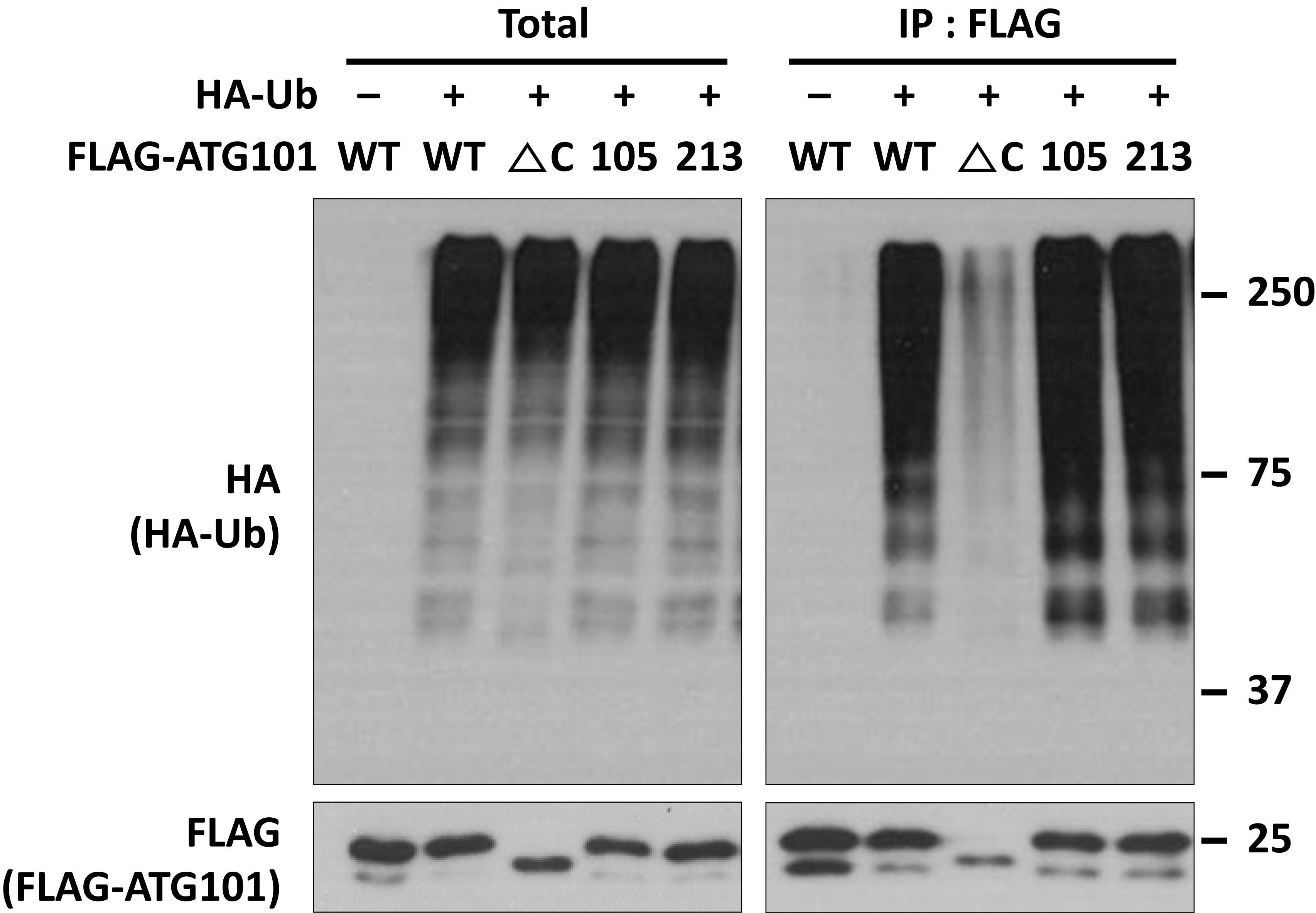

(b)

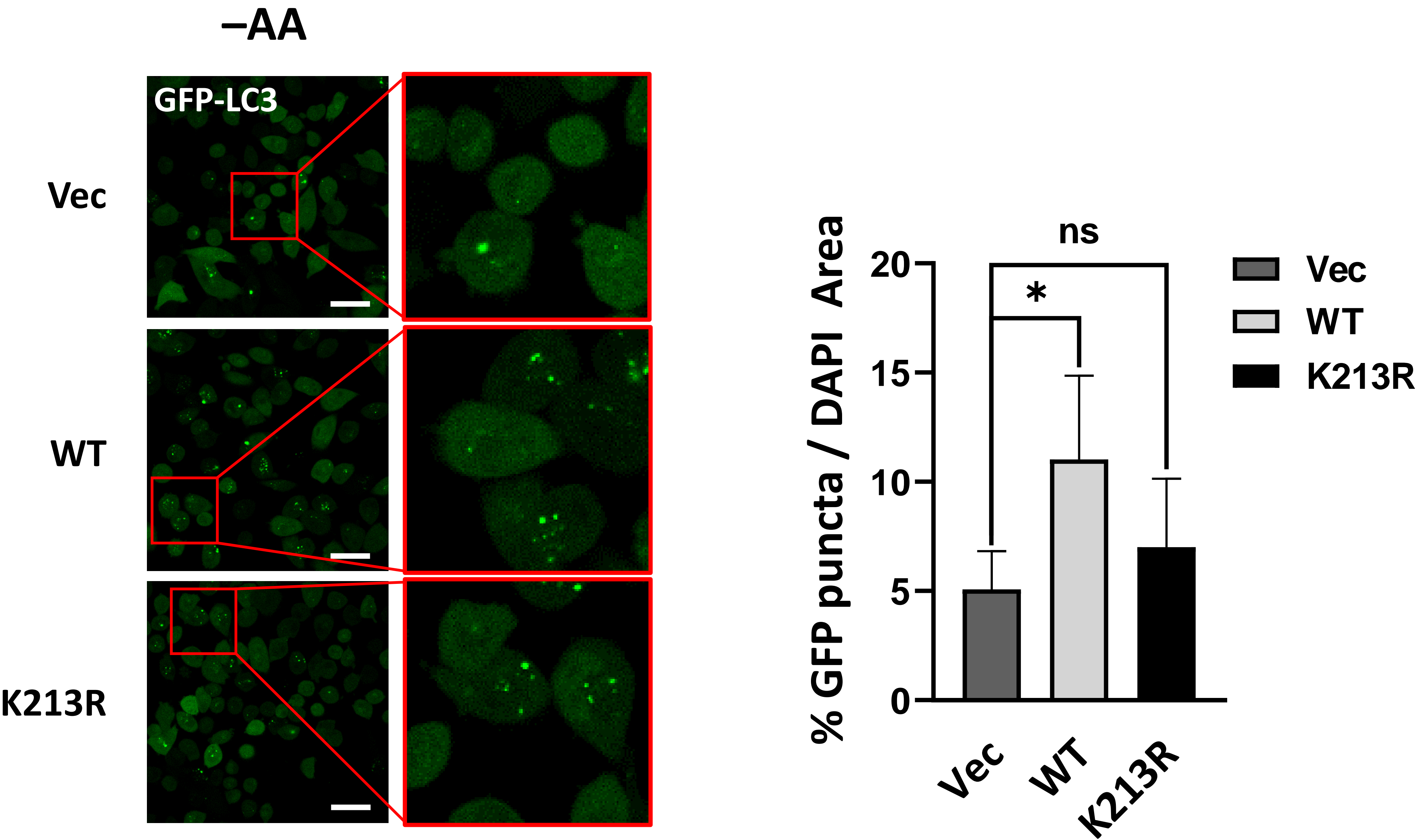

(c)

Supplement: Supplementary file 1 [file ijms-22-09182-s001.zip › Supplementary Figure S4.pdf]

Supplementary Figure S5

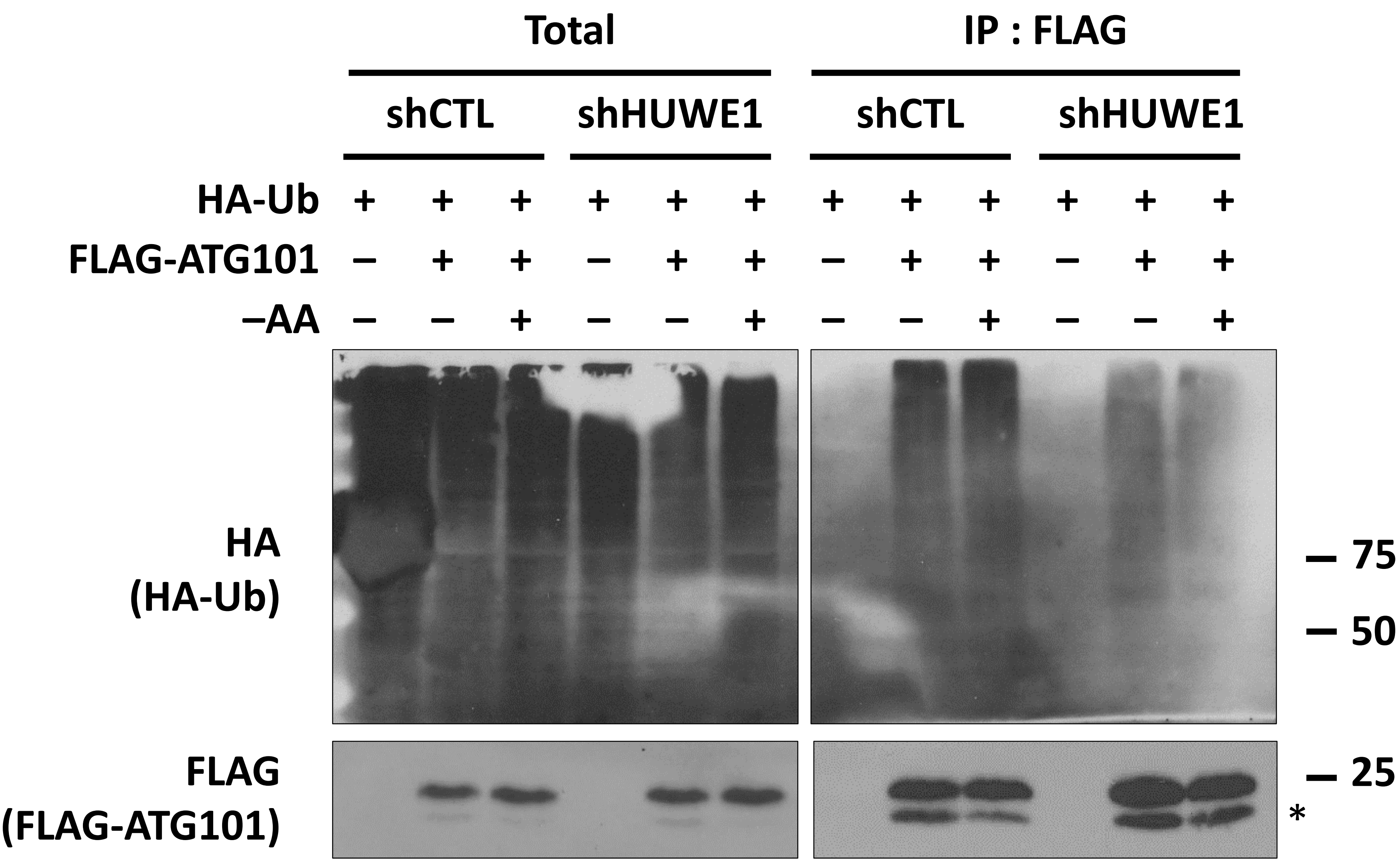

Supplement: Supplementary file 1 [file ijms-22-09182-s001.zip › Supplementary Figure S5.pdf]

Supplementary Figure S6

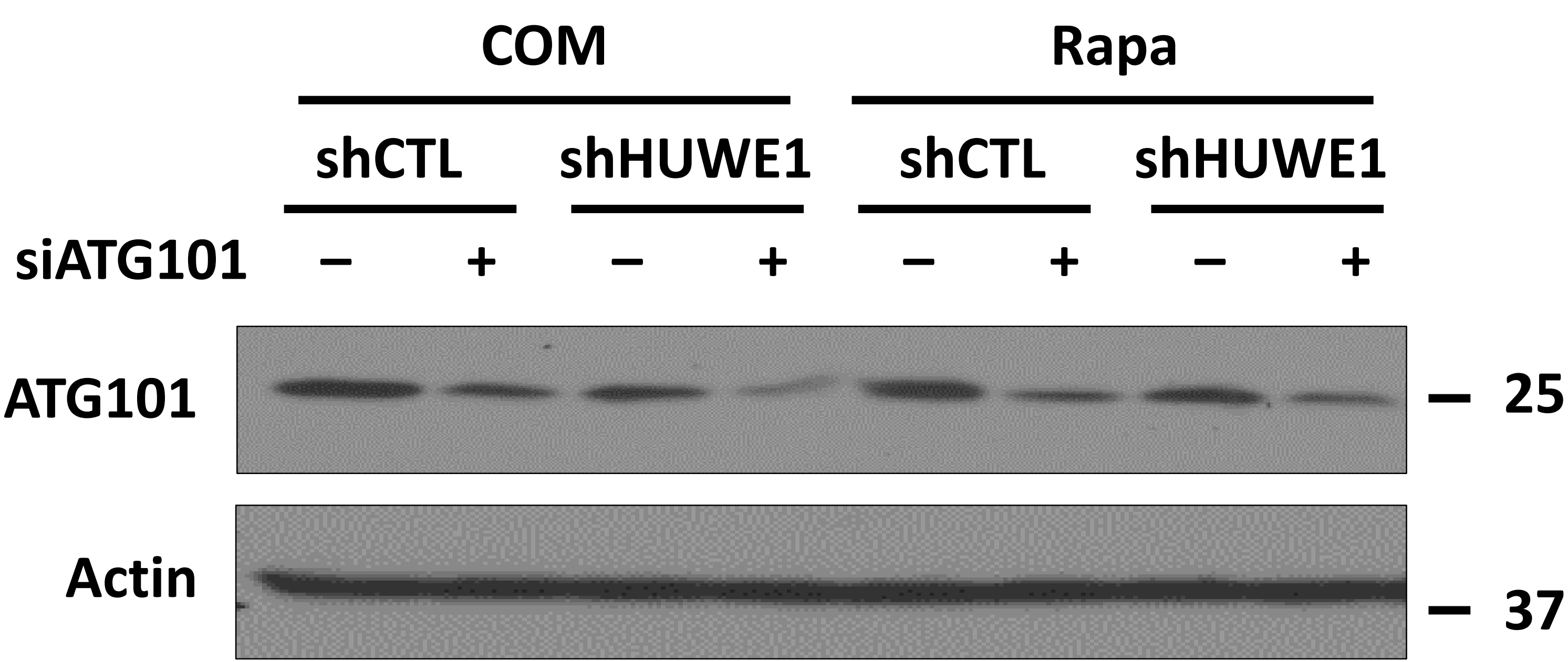

(a)

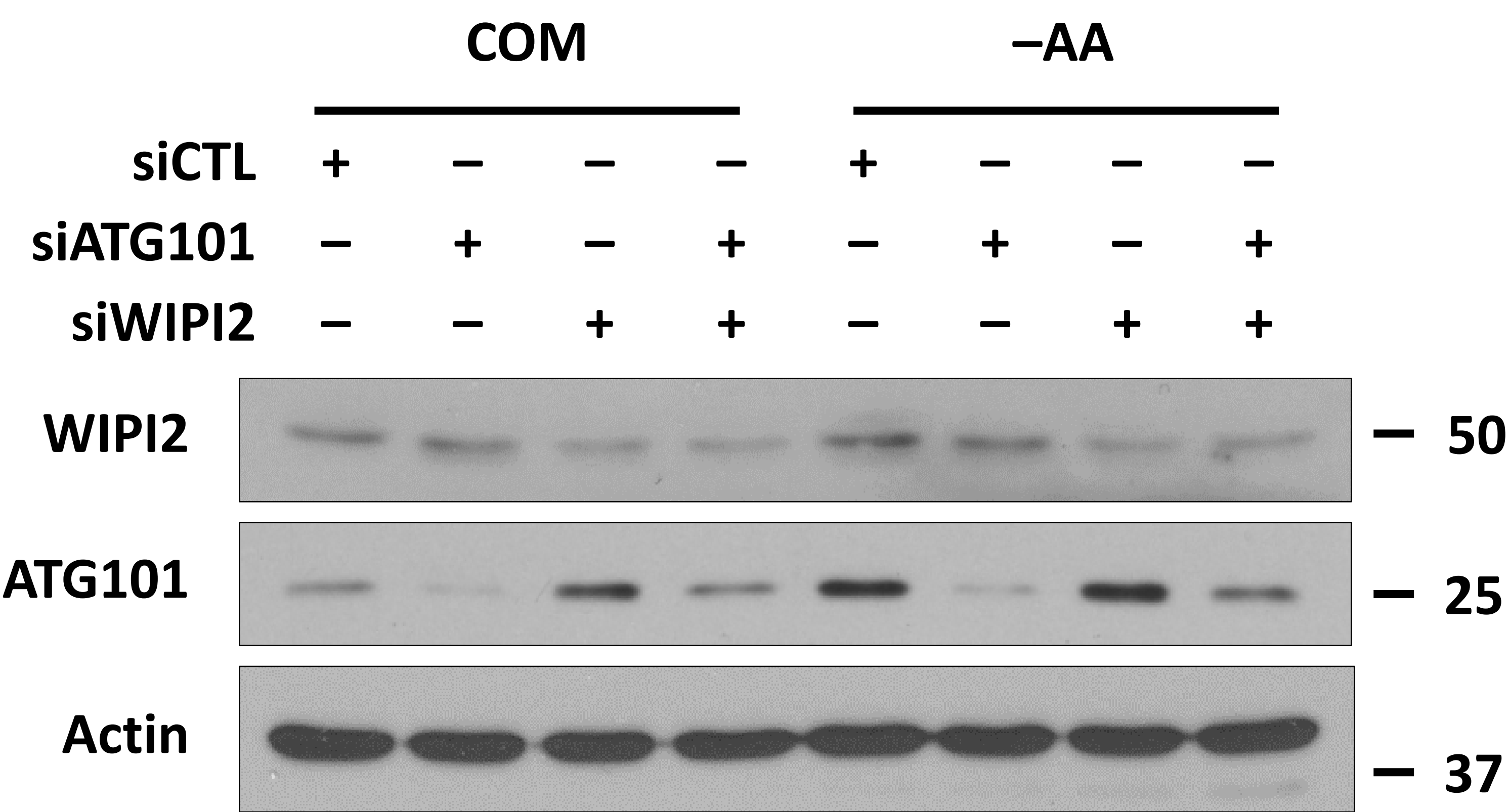

(b)

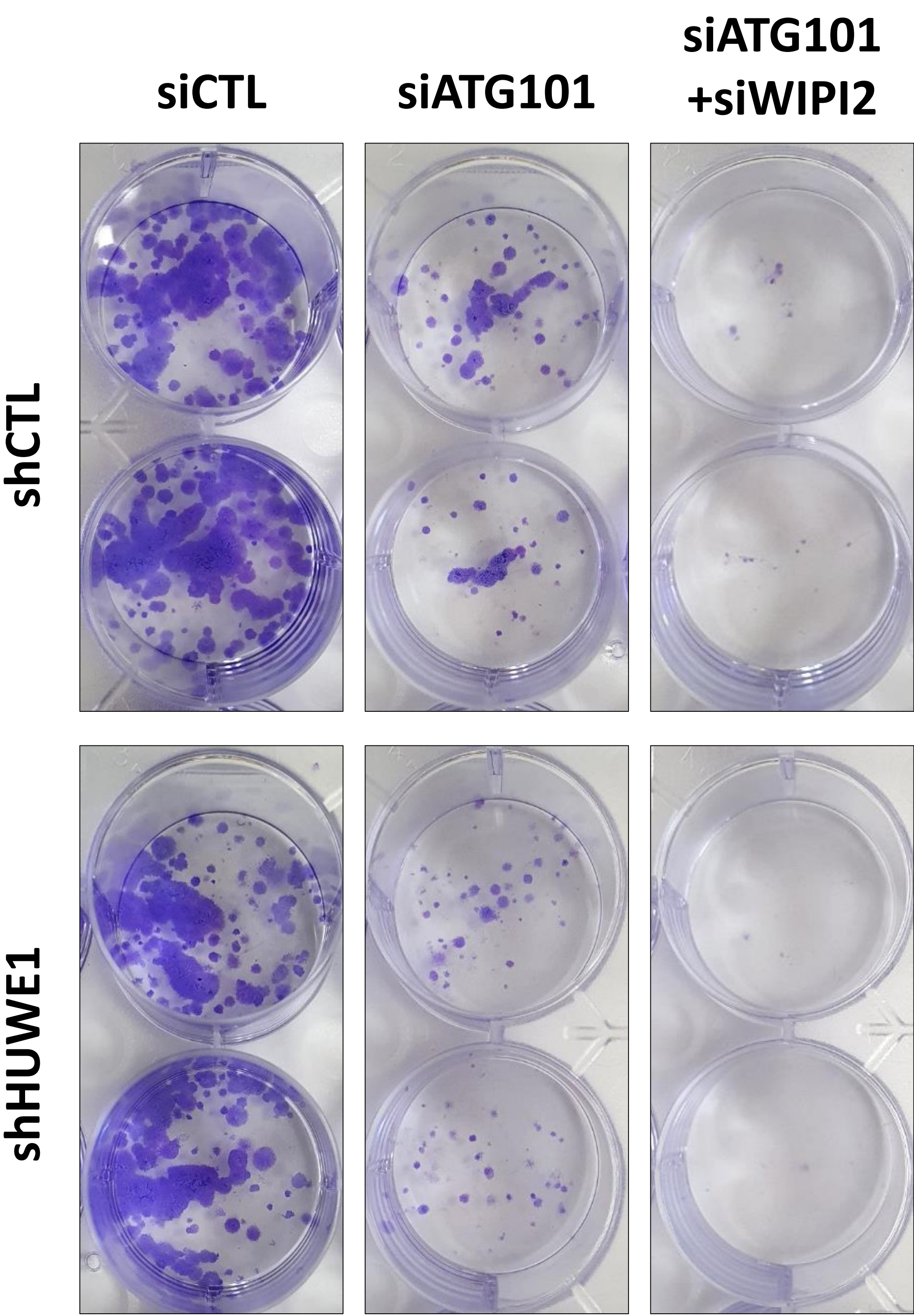

(c)

Supplement: Supplementary file 1 [file ijms-22-09182-s001.zip › Supplementary Figure S6.pdf]

## Slide 1
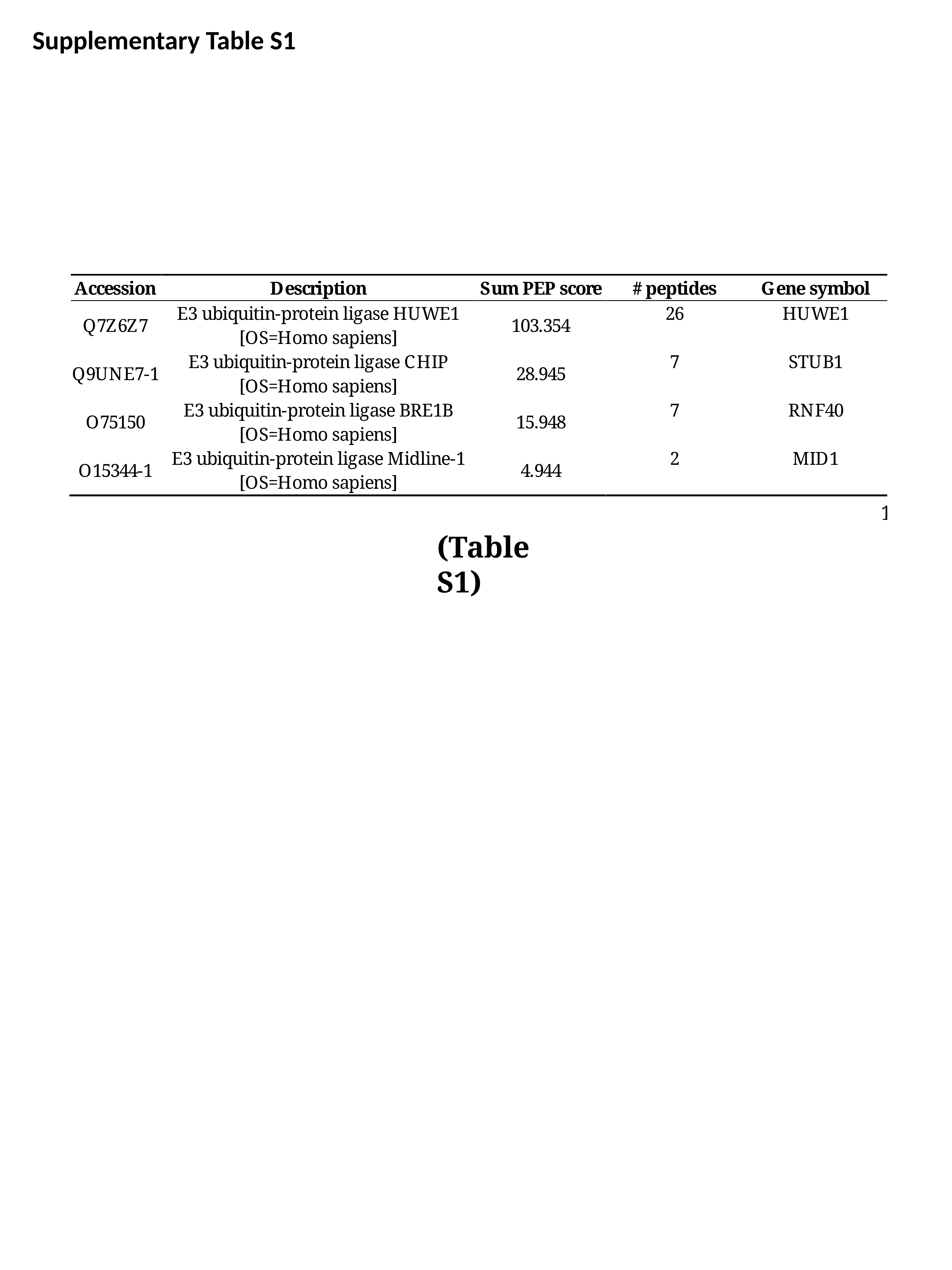

Supplementary Table S1
(Table S1)

Supplement: Supplementary file 1 [file ijms-22-09182-s001.zip › Supplementary Table S1.pptx]
